# Pandemic H1N1 Influenza Isolated from Free-Ranging Northern Elephant Seals in 2010 off the Central California Coast

**DOI:** 10.1371/journal.pone.0062259

**Published:** 2013-05-15

**Authors:** Tracey Goldstein, Ignacio Mena, Simon J. Anthony, Rafael Medina, Patrick W. Robinson, Denise J. Greig, Daniel P. Costa, W. Ian Lipkin, Adolfo Garcia-Sastre, Walter M. Boyce

**Affiliations:** 1 One Health Institute, School of Veterinary Medicine, University of California Davis, Davis, California, United States of America; 2 Department of Microbiology, Mount Sinai School of Medicine, New York, New York, United States of America; 3 Global Health and Emerging Pathogens Institute, Mount Sinai School of Medicine, New York, New York, United States of America; 4 Center for Infection and Immunity, Mailman School of Public Health, Columbia University, New York, New York, United States of America; 5 Laboratory Molecular Virology, Instituto Milenio en Inmunología e Inmunoterapia, Centro de Investigaciones Médicas y División de Pediatría, Facultad de Medicina, Pontificia Universidad Católica de Chile, Santiago, Chile; 6 Center for Ocean Health, Long Marine Laboratory, University of California Santa Cruz, Santa Cruz, California, United States of America; 7 The Marine Mammal Center, Sausalito, California, United States of America; 8 Department of Pathology, Immunology and Microbiology, School of Veterinary Medicine, University of California Davis, Davis, California, United States of America; The Ohio State University, United States of America

## Abstract

Interspecies transmission of influenza A is an important factor in the evolution and ecology of influenza viruses. Marine mammals are in contact with a number of influenza reservoirs, including aquatic birds and humans, and this may facilitate transmission among avian and mammalian hosts. Virus isolation, whole genome sequencing, and hemagluttination inhibition assay confirmed that exposure to pandemic H1N1 influenza virus occurred among free-ranging Northern Elephant Seals (*Mirounga angustirostris*) in 2010. Nasal swabs were collected from 42 adult female seals in April 2010, just after the animals had returned to the central California coast from their short post-breeding migration in the northeast Pacific. Swabs from two seals tested positive by RT-PCR for the matrix gene, and virus was isolated from each by inoculation into embryonic chicken eggs. Whole genome sequencing revealed greater than 99% homology with A/California/04/2009 (H1N1) that emerged in humans from swine in 2009. Analysis of more than 300 serum samples showed that samples collected early in 2010 (n = 100) were negative and by April animals began to test positive for antibodies against the pH1N1 virus (HI titer of ≥1∶40), supporting the molecular findings. In vitro characterizations studies revealed that viral replication was indistinguishable from that of reference strains of pH1N1 in canine kidney cells, but replication was inefficient in human epithelial respiratory cells, indicating these isolates may be elephant seal adapted viruses. Thus findings confirmed that exposure to pandemic H1N1 that was circulating in people in 2009 occurred among free-ranging Northern Elephant Seals in 2010 off the central California coast. This is the first report of pH1N1 (A/Elephant seal/California/1/2010) in any marine mammal and provides evidence for cross species transmission of influenza viruses in free-ranging wildlife and movement of influenza viruses between humans and wildlife.

## Introduction

Transmission of influenza A viruses among species is thought to be an important factor in the evolution and ecology of these viruses. To date there has been evidence for interspecies transmission between birds and marine mammals and seals and humans [1]–[3] as avian origin isolates (H4N5, H3N8) have been detected in harbor seals (*Phoca vitulina*) dying with pneumonia; and transmission to humans (H7N7) has been documented following exposure to infected seals that died with disease. Thus, these data suggest that seals can both become infected and transmit influenza viruses to conspecifics and other species.

Influenza A viruses have long been documented in marine mammals associated with outbreaks, including during the winter of 1979 to 1980 when H7N7 was isolated in harbor seals dying with severe viral pneumonia off the New England coast, again in 1982–1983 when H4N5 was isolated, and most recently in 2011 when H3N8 was isolated [1], [4]–[6]. However, continued surveillance since the first outbreak in 1979 has also resulted in isolation of H4N6 and H3N3 viruses from tissues from stranded seals when no increase in deaths was observed [7]. Two influenza A viruses (H13N2 and H13N9) have also been isolated from tissues from a sick pilot whale (*Globicephala meleana*) that died following a mass stranding event on the New England coast in 1984, however it was unclear if the influenza viruses played a role in the whale strandings [8]. Furthermore, serosurveys have documented widespread exposure globally to multiple HA (H3, 4, 6, 7, 10, 12) and NA (N2, 3, 7, 8) subtypes including in ringed (*Phoca hispida*), harp (*Phoca groenlandicus)*, and hooded (*Cystophora cristata*) seals, and walrus (*Odobenus rosmarus*), as well as more recently in harbor seals off California [9]–[12]. Given that exposure has been detected to multiple strains, co-infection in marine mammals may lead to reassortment and selection of mammalian adapted viruses. Infrequently, antibodies against influenza virus strains (H3N2) that circulated worldwide in humans have been detected in seals [13], [14], indicating that exposure to these human-adapted viruses may be sporadic and infection self-limiting in marine mammals.

Surveillance for influenza A viruses in more than 900 marine mammals from ten different species off the Pacific coast from Alaska to California from 2009 to 2011 also included serial sample collection from free-ranging juvenile and adult female Northern elephant seals (*Mirounga angustirostris*) when they came ashore and congregated for brief periods between biannual foraging migrations. Northern elephant seals dive continuously to forage at great depths when at sea (typically between 300 to 700 m but as deep as 1700 m) and females spend the vast majority of their lives ranging throughout the northeast Pacific and Gulf of Alaska, following preferred routes [15]. The two foraging trips consist of a short post-breeding migration (February to May) before returning to land for one month to molt; and a long post-molting migration while gestating (June to January), before returning to land for one month to give birth and breed on natal colonies [16]. After birth, new born pups remain on or close to shore for the first months of life learning to swim and forage. As a part of the Tagging of Pacific Predators (TOPP) program [17], adult Northern elephant seals were tagged each year to track at-sea movements and behavior, making them ideal for monitoring changes in exposure to and detection of new infections as during both time points on land as a subset of seals are handled and serially sampled. Here we describe the identification, isolation and characterization of pandemic H1N1 influenza virus in nasal swabs from two free ranging adult female Northern Elephant Seals. Sequence analysis indicated that the two isolates corresponded to the pandemic H1N1 isolate that was circulating in humans in 2009 and serologic analysis confirmed that exposure occurred in the population off the central California coast in the spring of 2010. This is the first confirmation of pH1N1 influenza infection in marine mammals.

## Materials and Methods

### Ethics Statement

This work was completed under National Marine Fisheries Service Marine Mammal permits # 786–1463, 87–143 and 932-1489-00. The animal use protocol for all procedures on free-ranging elephant seals was reviewed and approved by the University of California at Santa Cruz Institutional Animal Care and Use Committee and followed the guidelines established by the Canadian Council on Animal Care and the ethics committee of the Society of Marine Mammalogy.

### Animal Sampling

Nasal swabs and, when possible, paired serum samples were collected in 2010 and 2011 from juvenile (2–3 yrs, n = 8) and adult (5–13 yrs, n = 64) female Northern elephant seals that were captured at Año Nuevo State Reserve, San Mateo county, California, USA (37.116N, −122.331W) prior to leaving on their annual migration in February-March and upon their return from sea in April-May, either at Año Nuevo State Reserve or Pt. Piedras Blancas, San Simeon, CA, USA (35.667N, −121.278W). Contact between the seals and humans in both State Reserves was limited. Animals were captured as a part of ongoing studies and females were equipped with satellite tags and time-depth recorders (SPOT4, SPOT5, MK10-AF, and MK9; Wildlife Computers, Redmond, WA) prior to leaving shore to monitor at sea movement, diving and foraging behavior [15]. Nasal swabs were collected using a sterile-tipped applicator and placed into viral transport media (VTM). Samples were kept on ice prior to storage at −80°C until laboratory analysis.

Archived serum samples (n = 238) were obtained from January 2010 to May 2011 from Northern elephant seal pups (1–3 months old) collected upon admission to The Marine Mammal Center, a rehabilitation center, in Sausalito, CA, USA to further evaluate the timeline and geographic extent of exposure to influenza. Elephant seal pups, following birth at natal colonies and their mothers’ return to sea, stranded at 125 different locations in nine counties along the central California coast from San Luis Obispo County in the south to Mendocino County in the north spanning approximately 600 km of coastline. Stranded pups were rescued on beaches and admitted for rehabilitation and likely had limited close contact with humans until admitted to the rehabilitation center. The Chi-square test of independence was used to assess differences in prevalence of exposure in adults and pups and within age classes by year.

### Virus Detection and Isolation

VTM from nasal swab samples was screened for the presence of influenza A matrix (M) gene using two-step realtime RT-PCR [18]. Briefly, RNA was extracted from each swab sample using the MagMAX-96 Viral RNA Isolation Kit (Ambion, Austin, Texas). cDNA was synthesized using the M-MLV reverse transcriptase enzyme (Invitrogen, Carlsbad, California) and random hexamers (Invitrogen) and screened by rRT-PCR targeting the matrix gene [19]. Virus isolation was performed on positive samples by inoculating 100 µl of VTM into two 9 to 11 day old SPF embryonated chicken eggs (Charles River, North Franklin, Connecticut) by standard methods. The eggs were candled daily to monitor for embryo mortality. Two passages were performed and allantoic fluid was tested for the presence of the avian influenza matrix gene after each passage [18].

### Sequence Analysis

RNA was extracted from allantoic fluid from egg passage two at Mount Sinai School of Medicine using the QIAamp Viral RNA minikit (Qiagen Inc., Valencia, CA) for initial sequencing. The eight viral fragments were amplified using a set of pH1N1 2009 specific primers (primers available upon request) and PCR products were sequenced by conventional Sanger sequencing. Total RNA from VTM and allantoic fluid aliquots from egg passage one was then also extracted for unbiased high-throughput pyrosequencing analysis to confirm the presence of and compare sequences of A (H1N1)pdm09 in positive samples at the Center for Infection and Immunity at Columbia University. cDNA was generated using Superscript II RT (Invitrogen, Carlsbad, CA, USA) and random octamers linked to a defined arbitrary, 17-mer primer sequence tail (MWG Huntsville, AL, USA). After RNase H treatment cDNA was amplified by the polymerase chain reaction (PCR), applying a 9∶1 mixture of the defined 17-mer primer sequence and the random octamer-linked 17-mer primer sequence, respectively. Products of 70 base pairs (bp) were selected by column purification (MinElute, Qiagen, Hilden, Germany) and ligated to specific linkers for sequencing on the 454 Genome Sequencer FLX (454 Life Sciences, Branford, CT, USA) without DNA fragmentation. Sequences were analyzed using software applications implemented at the GreenePortal website (http://tako.cpmc.columbia.edu/Tools/).

For the phylogenetic analysis 250 concatenated coding sequences from pandemic H1N1 isolates were selected from an alignment of 1442 sequences obtained from the NCBI influenza database (kindly provided by Dr. Vijaykhrisna, Singapore). The sequences represented each three month time period from April 2009 to March 2011 and were chosen randomly within each time period. Sequences were added manually from the earliest isolate (A/LaGloria-3/2009) to the two recent elephant seal isolates and included six pH1N1 sequences isolated from non-human hosts (five swine, one turkey). The alignment was manually inspected and corrected using Bioedit (v7.1.7 Ibis Biosciences, Carlsbad, CA, USA) and Geneious (Geneious Pro v5.3.6 software Biomatters Ltd., Auckland, New Zealand) software and used to estimate phylogeny and divergence times by Bayesian Markov chain Monte Carlo (MCMC), using BEAST v1.6.1. [20], with a HKY+gamma substitution model and a strict molecular clock, as used previously to analyze and date the phylogenetic relationships of influenza viruses, including pH1N1 [21]–[23]. Fifty million generations, sampling every 5000 generations, was performed and results were analyzed using Tracer v1.5.

### Virus Characterization in Tissue Culture

To evaluate and compare the replication kinetics of the elephant seal isolates, growth curves were performed in Madin Darby Canine kidney (MDCK) and primary Human Tracheobronchial Epithelial (HTBE) cells [24] alongside three A(H1N1)pdm09 reference strains: A/California/04/2009, A/Mexico/4108/2009, and A/Netherlands/602/2009. The replication kinetics experiments were performed twice. MDCK cells were seeded in triplicate at a dilution of 10^6^ cells/well in 6-well plates, a day prior to infection. On the day of infection cells were washed twice with 2 ml of PBS and incubated with virus inoculum at an MOI of 0.001 PFU/cell in a final volume of 200 µl. After incubation for 1 hr at 37°C, the virus inoculum was removed and 2 ml of MEM containing 0.3% bovine albumin and 1 µg/ml TPCK treated trypsin was added to each well. At 12, 24, 36, 48, 60, 72 and 84 hours post infection 200 µl of supernatant was removed for virus titration and replenished with same amount of fresh media. HTBE primary cultures were differentiated in an air-liquid interphase in dual-chamber 12 well plates, as previously described [20]. Before infection (performed in triplicate), cell monolayers were washed 10 times with PBS to remove mucus produced by the cells and virus inoculum was added at a MOI of 0.001 PFU/cell. At the indicated time points 200 µl of PBS was added to the cells, incubated for 15 minutes and harvested for viral titration. The viral titers were determined by plaque assay. Growth curves were examined showing the mean viral titers and standard deviation of triplicate wells using Graphpad Prism software (GraphPad Software, Inc. La Jolla, CA, USA).

### Hemagglutinin Inhibition (HI) Assay

Serologic testing was performed on serum samples collected from the free-ranging adult and juvenile seals, and from pups upon admission to The Marine Mammal Center. The HI assay was performed as previously described [25], [26]. Briefly, elephant seal sera were inactivated by trypsin-heat-periodate treatment by mixing half a volume of trypsin 8 mg/ml (Sigma-Aldrich) in 0.1 M phosphate buffer, pH 8.2, with one volume of sera and the samples incubated for 30 min at 56°C. The samples were cooled to room temperature (RT), mixed with three volumes of 0.11 M metapotassium periodate and incubated at RT for 15 min. The samples were then mixed with three volumes of 1% glycerol saline and incubated for 15 min at RT. Finally, the samples were mixed and incubated with 2.5 volumes of 85% saline to dilute the samples to a concentration of 1∶10. After treatment 25 µl aliquots of 2-fold serially diluted serum samples were incubated with 25 µl of virus containing 8 HA units of influenza virus A/Netherlands/602/2009 H1N1 (NL/602) and a subset of serum samples (n = 150) were also incubated with influenza virus A/Brisbane/10/2007 H3N2 (Br/10) and A/Brisbane/59/2007 H1N1 (Br/59). Incubation was at 4°C for 30 min, followed by incubation with 50 µl of 0.5% turkey (for NL/602 and Br/10) or chicken (for Br/59) red blood cells (Lampire Biological Laboratories) at 4°C for 45 min. The HI titer was defined as the reciprocal of the highest serum dilution that inhibited hemagglutination and a titer >40 was used as the cut-off value for determining seropositive samples.

## Results

### Detection of A(H1N1)pdm09-like Infection in Northern Elephant Seals

**Figure 1:**
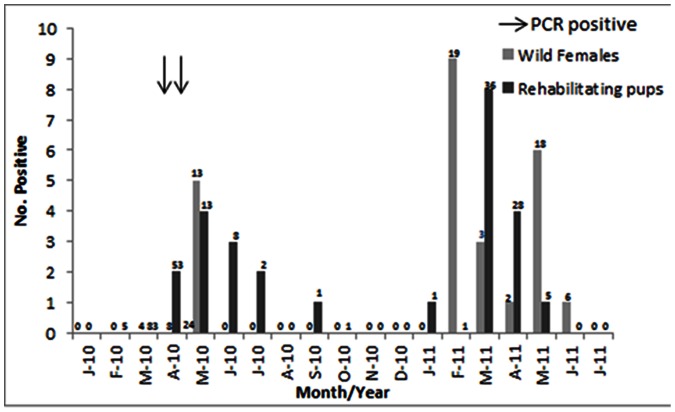
Evidence of exposure to pandemic H1N1 followed by seroconversion in Northern elephant seals (*Mirounga angustrostris*) off central California in 2010 to 2011. The arrows indicate when infected seals were detected by PCR and virus isolation, the bars represent the number of free-ranging adult females (grey bars) and pups upon admission to rehabilitation (black bars) that tested positive for antibodies (numbers above the bars were the number of seals tested each month).

**Figure 2:**
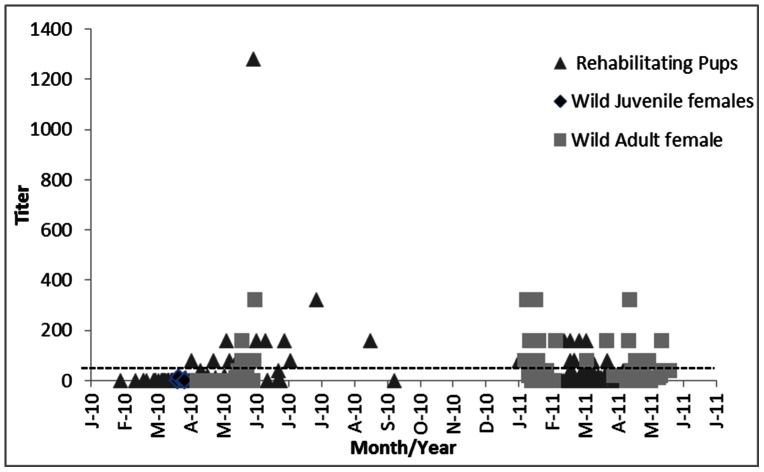
Individual Hemagglutinin Inhibition Assay serologic results over time showing positive titers that ranged from 1∶80 to 1∶1280, but varied by animal group in 2010 to 2011. The line represents the cut-off value for seropositive samples of titers ≥40.

Free-ranging juvenile Northern elephant seals (n = 8) were sampled upon tag deployment at Año Nuevo State Reserve from 22 to 28 March 2010; and adult females sampled upon return from sea (n = 33) at Año Nuevo State Reserve and Pt. Piedras Blancas from 8 April to 31 May 2010, upon tag deployment (n = 24) from 1 February to 31 March 2011 and again upon return from sea (n = 29) from 17 April to 13 June 2011 (16 animals were sampled at two or three time points). In 2010 all free-ranging animals tested negative for the matrix gene by RT-PCR (n = 17) until 30 April when the first adult female (M778) tested positive upon return (within 4 days) to Piedras Blancas (Figure 1). The second female (WX541) tested positive on 5 May upon her return (within 3 days) to Año Nuevo (Figure 1). Four seals handled and sampled between 30 April and 6 May, and another 21 sampled after 6 May all tested negative for the matrix gene by RT-PCR. All free-ranging seals tested were negative for antibodies to pandemic H1N1 until 19 May 2010, after which seropositive animals were detected at both Año Nuevo and Piedras Blancas (Figure 1). Positive HI titers ranged from 1∶80 to 1∶320 and 14% of the seals tested seropositive (Figure 2). No free-ranging elephant seals tested positive for the matrix gene by RT-PCR in 2011 but 16 animals (40%) were seropositive for antibodies to pandemic H1N1. None of the free-ranging seals tested positive for antibodies to either the seasonal H1N1 or H3N2 isolates that were circulating in people in California in 2010.

Archived serum samples tested were collected from Northern elephant seal pups admitted for rehabilitation from 29 January to 6 October 2010 (n = 167) and 28 January to 16 May 2011 (n = 71). None of these samples tested positive for antibodies to pandemic H1N1 until 4 April 2010 and the number of seropositive animals increased over the following months (Figure 1), with 12 (7%) pups testing positive. Positive HI titers ranged from 1∶80 to 1∶1280 (Figure 2). Since samples were collected upon admission to the rehabilitation center, HI titers reflected exposure to pH1N1 prior to entry to the rehabilitation center. Interestingly 14 pups (19%) also tested seropositive in 2011, but positive titers were lower overall and ranged from 1∶80 to 1∶160 (Figure 2). The proportion of adults (14%, 5/36) and pups (7%, 12/154) that tested seropositive in 2010 was similar (p = 0.19). However, although the proportion of both age classes increased in 2011 (P<0.01), more adults (40%, 20/44) had detectable antibodies compared to pups (19%, 14/71) (P<0.01).

### Northern Elephant Seal Movements at Sea

**Figure 3:**
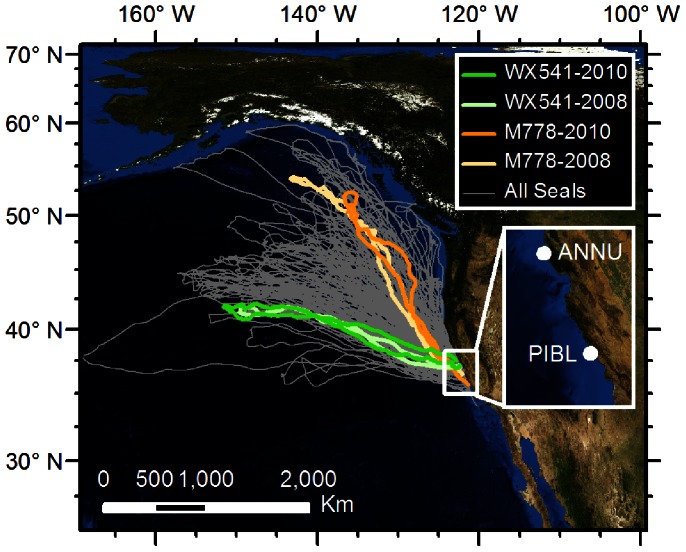
Tracking data from two adult female Northern elephant seals (*Mirounga angustrostris*) that tested positive for A(H1N1)pdm09 infection (WX541 in green, M778 in orange), showing both their tracks in 2008 and 2010 leaving from the Año Nuevo, CA, USA colony and returning to either Año Nuevo or Piedras Blancas colonies. Tracks from other adult female Northern elephant seals movement tagged between 2004 and 2010 shown in grey for comparison. Light blue areas represent the waters on the continental shelf and the dark blue represents deep pelagic waters.

Both adult seals that tested PCR positive were captured and instrumented with satellite tags in February 2010 at Año Nuevo State Reserve. M778 was born in 2002 and WX541 in 2001, both had also been previously instrumented in 2008. As expected both seals made similar trips in both years. M778 left Año Nuevo on 11 February went north and travelled off the continental-shelf from California to southeast Alaska and foraged off the shelf in pelagic waters (Figure 3). She returned to Piedras Blancas on 24 April 2010. WX541 left Año Nuevo on 8 February and also went north but travelled to and foraged in the mesopelagic zone in the northeast Pacific (Figure 3). She returned to Año Nuevo on 6 May 2010. When comparing the movements of these two elephant seals to data collected from more than 200 female Northern elephant seals from 2004–2010 (Figure 3), movements of these two were consistent with the other animals showing they travelled through and used the entire region of the northeast Pacific, Gulf of Alaska and along the Aleutian Islands but concentrated their foraging efforts along a narrow band at the boundary between the sub-arctic and sub-tropical gyres [15].

### Isolation and Genomic Analysis of H1N1 Influenza from Northern Elephant Seals

Virus isolates were obtained from nasal swab samples from both adult female seals that tested RT-PCR positive for the M gene after inoculation into embryonated chicken eggs. These isolates were sequenced after the second passage and showed greater than 99% homology for all segments to pandemic influenza A/California/04/2009 [27] that circulated in people in California in 2009. In accordance with conventional nomenclature, the virus isolates were named A/elephant seal/California/1/2010 and A/elephant seal/California/2/2010 and the sequences submitted to GenBank and assigned accession numbers (JX865419 to JX865426 and KC222499 to KC222506). The nucleotide sequences of both isolates were almost identical to each other, as Segments 1 (PB2), 2 (PB1), 4 (HA), 5 (NP) and 7 (M) were identical; and Segments 3 (PA), 6 (NA) and 8 (NS) each had one nucleotide difference but all were silent changes. A total of 24 amino acid changes were found when comparing translated sequences to A/California/04/2009; 7 changes in the HA gene, five changes in the NA gene, four in the NP gene, two changes in the NS gene, three changes in the PA gene, two changes in the PB1 gene and one change in the PB2 gene (Table 1).

**Table 1:** Amino acid substitutions in the elephant seal pH1N1 strains (A/elephant seal/California/1/2010) isolated in California, USA, in 2010 compared with A/California/04/2009.

Virus	GenBank #	PB2 (Seg 1)		GenBank #	PB1 (Seg 2)			
A/California/04/2009	FJ966079	E (120)		FJ966080	A (643)	K (736)		
M778		D			T	G		
WX541		D			T	G		
Virus	GenBank #	PA (Seg 3)						
A/California/04/2009	FJ966081	V (14)	P (224)	N (359)				
M778		I	S	I				
WX541		I	S	I				
Virus	GenBank #	HA (Seg 4)						
A/California/04/2009	FJ966082	P (100)	S (145)	T (214)	S (220)	D (239)	I (338)	E (391)
M778		S	P	A	T	N	V	K
WX541		S	P	A	T	N	V	K
Virus	GenBank #	NP (Seg 5)						
A/California/04/2009	FJ966083	V (100)	D (101)	A (260)	I (365)			
M778		I	N	T	V			
WX541		I	N	T	V			
Virus	GenBank #	NA (Seg 6)						
A/California/04/2009	FJ966084	V (81)	V (106)	N (248)	N (386)	E (398)		
M778		I	I	D	S	D		
WX541		I	I	D	S	D		
Virus	GenBank #	NS (Seg 8)						
A/California/04/2009	FJ966086	E (97)	I (123)					
M778		A	V					
WX541		A	V					

In order to confirm the presence of pH1N1 in the samples and to compare sequences, whole genome sequences were also amplified from RNA extracted from the VTM and passage 1 allantoic fluid samples through unbiased high throughput sequencing. Results confirmed the presence of pH1N1 in both samples. The sequences derived from the VTM and passage 1 samples from both isolates were identical to each other and included the same nucleotide differences between the two isolates that resulted in silent changes as described above. They were also mostly identical to the sequences obtained from the pass 2 allantoic fluid samples with three silent nucleotide changes found in the HA, NP and PB1 genes.

**Figure 4:**
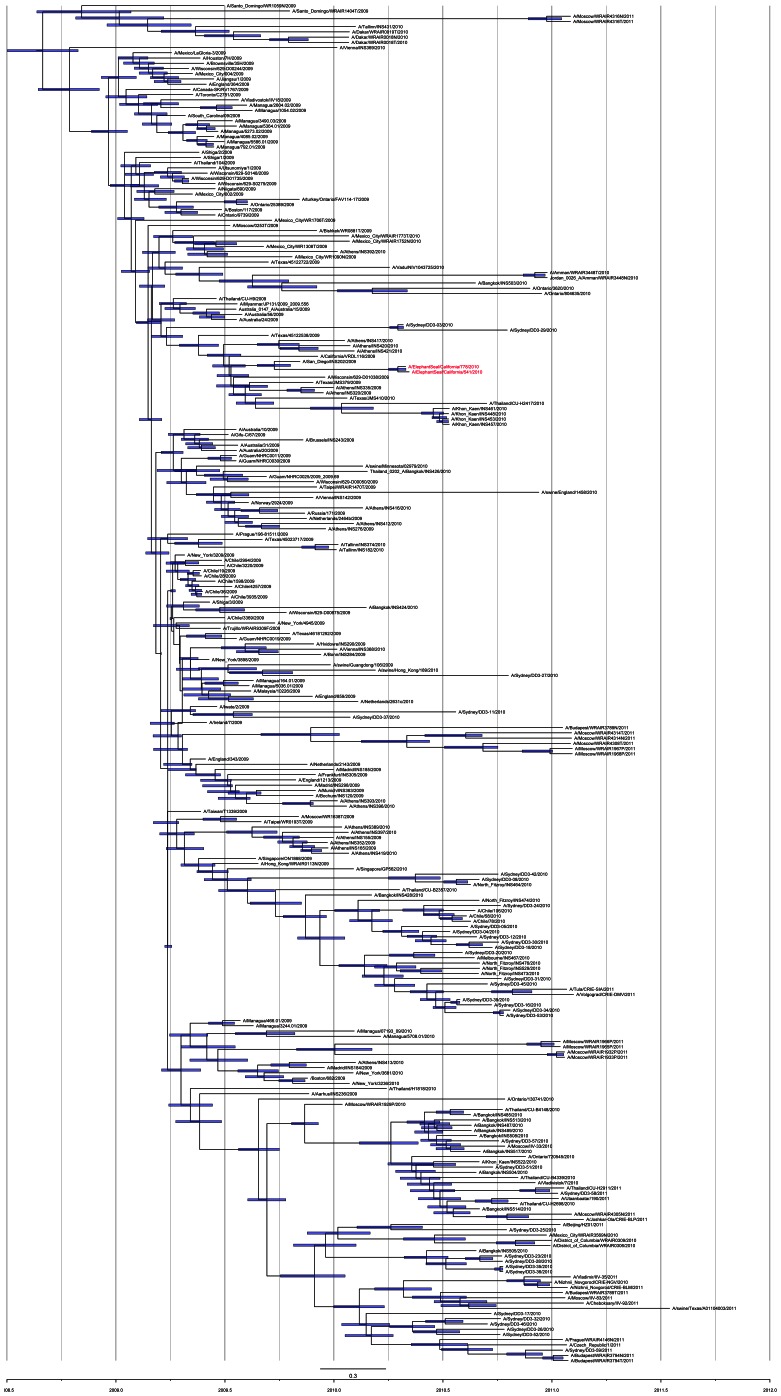
Dated phylogenetic tree of the relationship of the two Northern elephant seal isolates (shown in red) relative to other human and non-human pH1N1 isolates, showing the closest isolate was A/San Diego/INS202/2009 in humans. The estimated time to most recent common ancestor (TMRCA) 95% Bayesian credible intervals are indicated.

Bayesian analysis to estimate the phylogenetic relationship of the Elephant Seal isolates with a selection of more than 250 influenza pH1N1 whole genome sequences showed the two NES viruses clustered together but were closely related to human pH1N1 isolates, and confirmed they were derived from the pandemic virus (Figure 4). The time to most recent common ancestor (TMRCA) was estimated at April 17, 2010 (95% Bayesian credible interval between April 1 and April 30). The TMRCA between the NES isolates and the closest human isolate (A/San Diego/INS202/2009) was estimated at September 22, 2009 (95% Bayesian credible interval Aug/23 to Oct/19).

### Northern Elephant Seals H1N1 Isolates Attenuated in HTBE Cells in vitro

**Figure 5:**
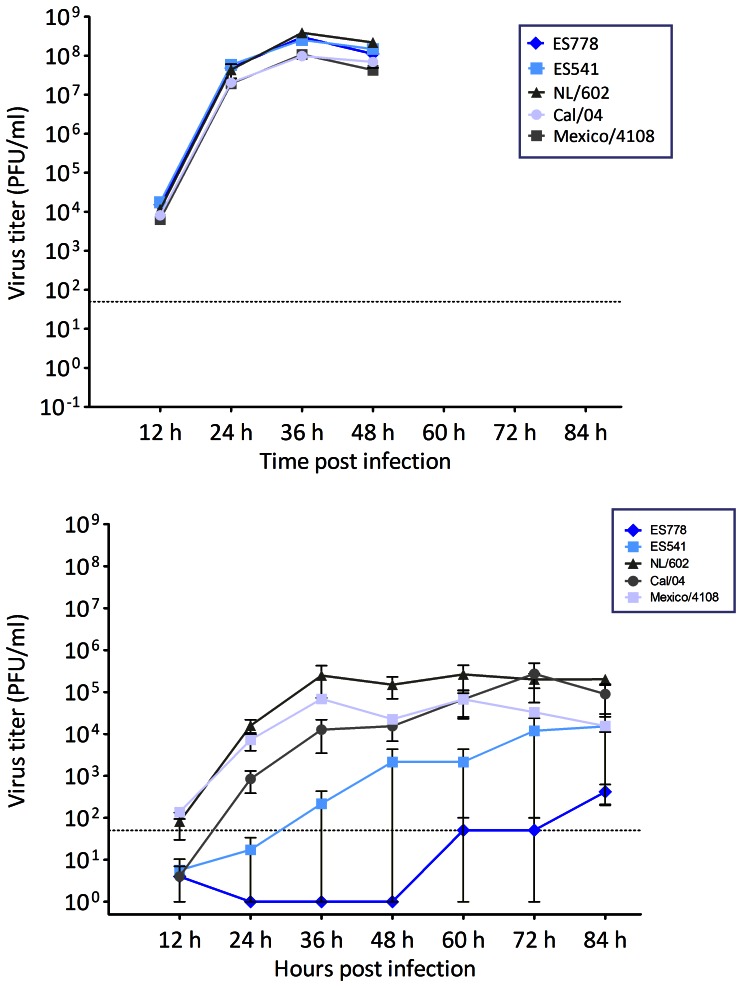
In vitro replication kinetics of A(H1N1)pdm09-like viruses isolated from Northern elephant seals showing growth curves of the two elephant seal pH1N1 isolates in Madin Darby Canine kidney (MDCK) cells (A) and Human Tracheobronchial Epithelial (HTBE) cells (B) compared to three reference stains: A/California/04/2009, A/Mexico/4108/2009, A/Netherlands/602/2009. Supernantants were collected at the indicated time points and titrated by standard plaque assay, graphs show the mean titres of triplicate wells per time point and error bars indicate the standard deviation. The dotted line represents the limit of detection of 50 PFU/m.

Viral growth kinetics of the two elephant seal isolates performed in MDCK and HTBE cells and compared to reference pandemic H1N1 strains showed that growth in MDCK cells was comparable to the three reference strains over a 48-hour infection period (Figure 5a). Although all of the isolates produced variable growth curves and less detectable virus in the HTBE cells, both elephant seal isolates replicated less efficiently compared to all of the human reference strains in the human primary cell cultures, as their replication kinetics was slower and no detectable virus was produced consistently until 36–48 h post infection (Figure 5b). Whereas the A(H1N1)pdm09 human reference strains readily produced viral progeny within the first 24 h post infection (Figure 5b). The isolate from ES541 replicated slightly faster than the isolate from ES778 in the first experiment (Figure 5b) while the opposite occurred in the second experiment (data not shown). Results indicated the difference was likely due to experimental variation rather than a real difference between the two isolates, as all of the virus produced by the isolate from ES541 up to 48 h post infection occurred in only one of the triplicate wells.

## Discussion

This is the first report of pandemic H1N1 detected in any marine mammal. It is unclear how and when exposure occurred, but it is possible that exposure to the virus may have occurred when the animals were at sea. Given that samples from all other species tested negative for influenza A, results indicated that widespread exposure did not occur among marine mammals.

Virus was not detected in any adult females until after females began to return from sea in April 2010 nor did any adult females or pups test positive for antibodies until April 2010 after females collectively began returning to land (more than 100 pups tested negative from February to April prior to the first seropositive animal). The RT-PCR positive adult female seals were detected within days of each other (April 30 and May 5) at two different locations along the California coast separated by more than 200 miles, and both females were sampled within three or four days of returning to shore. Exposure on land would have required two separate exposure events at distant locations along the coast, which seems unlikely. Both the occurrence of infection at multiple sites along the coast and the short timeframe following return to shore and detection of virus supports exposure in these seals prior to reaching land, either while at sea or upon entering the near-shore environment, rather than upon returning to shore in April 2010, however the latter can’t be ruled out. Additionally, as the females concentrate along a narrow band to forage while at sea, the potential for a common exposure during their time at sea is possible. If the virus was circulating in the near-shore environment in the beginning of April then introduction may have occurred in one or more females upon returning from sea, prior to arrival on land. As adult females were already collectively returning from sea at the beginning of April and not all females were sampled for testing this seems possible, allowing for further spread following contact with other seals once on land. Alternatively it is possible that the virus was circulating in the near-shore or on-shore environment prior to the adult females returning to shore. This hypothesis may be supported by the detection of seropositive pups at The Marine Mammal Center prior to the first virus-positive adult female, thus viral transmission may have been occurring on land as early as the beginning of April 2010.

When at sea, elephant seals spend most of their time foraging concentrated in the northeast Pacific Ocean off the continental shelf in the mesopelagic zone, a highly productive region where many marine species and fisheries converge [28], [29]. Direct contact between humans and elephant seals seems unlikely in this remote region, however since A(H1N1)pdm09 has been detected in stool samples of hospitalized patients [30], it may be possible that exposure could have occurred through feces discharged from the large number of shipping vessels at sea traversing this area. Alternatively, exposure may have occurred through contact with other marine species, such as aquatic birds, that have been thought to be reservoirs for other influenza viruses [31], [32].

Phylogenetic analysis of the whole genome sequence showed the elephant seal isolates clustered together but were closely related to human pH1N1 isolates. The time to most recent common ancestor (TMRCA) was estimated at April 17, 2010, very close to the isolation date in this study. The TMRCA between the NES isolates and the closest human isolate (A/San Diego/INS202/2009) was estimated at September 22, 2009, suggesting that the introduction of the pandemic H1N1 influenza virus into the Northern elephant seal population occurred between the fall of 2009 and the spring of 2010.

As evaluation of the elephant seal isolates in cell culture showed that viral replication and pathogenesis was indistinguishable from that induced by the reference strains in the MDCK cells, but was inefficient in the human epithelial respiratory cells, results suggested these may be animal adapted viruses, as has been indicated previously in harbor seals with H7N7 infection [33]. Given the increased prevalence of pH1N1 specific antibodies measured over a short period of time, it is tempting to speculate that the virus was introduced into one or a small number of elephant seals and quickly acquired adaptive mutations that allowed for replication and transmission in the elephant seal population while reducing replication fitness in human epithelial respiratory cells. Efforts to identify the mutations and biological mechanisms involved in this adaptation are currently ongoing.

Of the 24 amino acid changes found when comparing translated sequences to reference strain A/California/04/2009, only three had not been documented previously [34]–[38]: E398D in the NA gene, A260T in the NP gene and N359I in the PA gene. The significance of these changes is currently unknown. Of the 21 other changes found when compared to A/California/04/2009, only one mutation (I365V in the NP gene) has not been found in other pH1N1 isolated from humans, and therefore most mutations cannot be considered to be elephant seal specific. The greatest number of changes was in the surface proteins, all seven changes in the HA gene and four of the five in the NA gene were common for late variant strains isolated in the Fall of 2009 [39]. Four amino acid changes found in other genes were also of interest: the E97A mutation was present in the NS1 gene which is thought to interfere with activity of the TRIM25 molecule resulting in disruption of the IFN-mediated innate immunity potentially, which may correlate with viral pathogenesis [34], the first documentation of this mutation occurring naturally in a host as to date it has only been produced in laboratory strains through reverse genetics; E120D in the PB2 gene, is not a common mutation, found in both elephant seal isolates, but has been documented in people by the Department of Defense Global Influenza Surveillance program [40]; the mutation found at D101N in the NP gene occurs more frequently in isolates from animals and can also be replaced with glutamic acid or glycine instead of asparagine, as occurred in the elephant seal isolates [41]; finally I365V in the NP gene is not a common mutation and found in only a few avian and swine-origin influenzas [42].

Interestingly, although no virus was detected by RT-PCR in any of the seals tested in 2011, antibodies to pandemic H1N1 were detected in pups born in 2011. Titers were lower in the pups tested in 2011 (1∶80 to 1∶160) compared to in 2010 (1∶80 to 1∶1280), and may represent passively transferred maternal antibodies as commonly occurs in other neonates [43]. Declining maternal antibodies to other pathogens have been measured in other seal species, such as to phocine herpesvirus in harbor seals [44], [45], but the pups in those studies were much younger (one to three weeks of age). Since elephant seal pups would not have immunologic memory from previous exposure to A(H1N1)pdm09 during 2010, as the adult seals would have, it seems plausible that maternal antibodies were detected. Since the timeline of maternal antibody decline is not currently known for elephant seal pups, and serial samples were not tested to determine if antibodies were declining in these pups, additional work is ongoing to further explore this possibility.

None of the sera analyzed were positive for antibodies against other influenzas that were circulating in people in California in 2010, including the 2010 seasonal H1N1 and H3N2 isolates. Given that the proportion of adults and pups that tested seropositive in 2010 was similar, and that antibodies were detected in all age classes during the month of April, these features are consistent with exposure to a new virus in the population at this time. Additionally, as follow-up (data are not shown here), nasal swab samples from free-ranging juvenile and adult elephant seals in 2012 also tested PCR negative, but a similar proportion tested seropositive in 2012 (35%, 13/37) compared to 2011 (40%). Titers from these seals were also decreased compared to the previous two years, perhaps suggestive of an earlier exposure with currently waning antibody levels. Thus, as expected, these results suggest that exposure to human influenza A viruses is likely not an annual occurrence in seals.

Given that none of the seals that were handled on the beach appeared ill nor were influenza related lesions documented in elephant seals that died in rehabilitation in 2010, it seems that influenza infection was asymptomatic and the disease self-limiting. Importantly, this work highlights that marine mammals may be infected with zoonotic pathogens and not show clinical signs of illness, thus being asymptomatic carriers. This work also emphasizes the additional biosafety measures that people working with and around marine mammals should adopt to adequately protect themselves to prevent exposure to diseases that although may not cause illness in the seals, could be quite pathogenic in humans, as well as to prevent transmission of diseases people may carry to the animals they are handling.
